# Association between peripheral hsa-miR-4456 expression and plasma oxytocin in men with hypersexual disorder and healthy controls: a secondary cross-sectional analysis

**DOI:** 10.3389/fnins.2026.1946302

**Published:** 2026-10-14

**Authors:** Adrian E. Desai Boström, Andreas Chatzittofis, Peter Andersson, Josephine Savard, Katarina Görts Öberg, Stefan Arver, John N. Flanagan, Jussi Jokinen

**Affiliations:** 1 Center for Psychiatry Research, Department of Clinical Neuroscience, Karolinska Institutet, Stockholm, Sweden; 2 Stockholm Health Care Services, Region Stockholm, Karolinska University Hospital, Stockholm, Sweden; 3 Department of Clinical Sciences/Psychiatry, Umeå University, Umeå, Sweden; 4 Medical School, University of Cyprus, Nicosia, Cyprus; 5 Department of Clinical Neuroscience/Psychology, Karolinska Institutet, Stockholm, Sweden; 6 Center for Clinical Research Dalarna, Uppsala University, Falun, Sweden; 7 Department of Medicine, Karolinska Institutet, Karolinska University Hospital, Stockholm, Sweden; 8 Department of Laboratory Medicine, Clinical Physiology, Karolinska Institutet, Stockholm, Sweden

**Keywords:** compulsive sexual behavior disorder (CSBD), hypersexual disorder, microRNA, miR-4456, biomaker, oxytocin

## Abstract

**Introduction:**

MicroRNAs are candidate regulators and biomarkers of molecular processes relevant to behavior, but their relationships with neuroendocrine signaling in humans remain poorly characterized. Earlier studies in overlapping cohorts identified hypermethylation-associated downregulation of peripheral blood-derived hsa-miR-4456 in men with hypersexual disorder and implicated oxytocin-related pathways through *in-silico* target prediction; a subsequent study found higher plasma oxytocin in the same clinical context. Whether miR-4456 expression and oxytocin covary within the same individuals had not been examined.

**Methods:**

We performed a secondary cross-sectional analysis of baseline, pretreatment measurements from clinically characterized men with hypersexual disorder and healthy male controls. miR-4456 was measured by RT-qPCR in EDTA blood-derived material and plasma oxytocin by radioimmunoassay. The submitted pooled robust regression included 74 paired measurements; revised group-adjusted and interaction models included 73 complete cases (51 hypersexual disorder and 22 controls). miR-4456, but not oxytocin, was rank-based inverse-normal transformed using Blom's method.

**Results:**

In the submitted pooled model, higher miR-4456 was associated with lower plasma oxytocin (β = −4.039 pmol/L, 95% CI −6.149 to −1.930; *p* < 0.001). After adjustment for diagnostic group, the estimate remained negative but was attenuated (β = −1.522, 95% CI −3.376 to 0.333; *p* = 0.106). The control-specific estimate was −0.613 (95% CI −3.693 to 2.467; *p* = 0.696), the hypersexual-disorder estimate was −2.223 (95% CI −4.514 to 0.069; *p* = 0.057), and the interaction was not significant (*p* = 0.414). *Post hoc* sensitivity analyses were directionally consistent but imprecise.

**Conclusions:**

To our knowledge, this is the first human study to directly examine paired peripheral miR-4456 and plasma oxytocin measurements. Although diagnostic-group differences explained part of the pooled association, the paired observation connects previously separate epigenetic, expression, pathway-prediction, and neuroendocrine findings and identifies a focused hypothesis for independent replication. The cross-sectional peripheral measurements do not establish directionality, causation, or central nervous system regulation. Experimental studies are needed to determine whether miR-4456 has any functional influence on oxytocin-related biology.

## 1 Introduction

MicroRNAs (miRNAs) are small non-coding RNAs that regulate gene expression by binding to the 3′-untranslated region (3′-UTR) of target messenger RNA (mRNA). This binding leads to mRNA degradation or repression of its translation. The biogenesis of miRNAs is spatially and temporally controlled, which results in their tissue-specific expression. Over 1,917 human precursors and 2,654 mature miRNAs have been described, and some of them have been associated with human pathologies (Rupaimoole and Slack, 2017). miRNAs are emerging as important regulators of normal and pathological behaviors, with the potential to serve as master regulators of many cellular processes (Wang et al., 2021). They have been shown to regulate the expression of more than half of protein-coding genes (Friedman et al., 2009) and certain miRNAs are capable of regulating several hundred different genes (Issler and Chen, 2015), making them unique regulators of biological and cellular pathways.

While miRNAs are best known for their intracellular functions, they can also be found in blood cells and in extracellular spaces such as plasma and serum (Cortez et al., 2011). The mechanisms by which miRNAs enter circulation are only partially understood, but evidence suggests that they may be released through apoptosis or necrosis or actively secreted from living cells through lipid structures or protein-miRNA complexes. The detection of miRNAs in body fluids not only provides insights into the physiological and pathological state of originating cells but also suggests a potential hormone-like role for these molecules as secreted gene regulators. There is accumulating evidence for the role of secreted miRNAs in intercellular crosstalk and for horizontal transfer of miRNAs, suggesting the possibility that circulating miRNAs may influence the function of adjacent or distant neuronal populations (Lakhal and Wood, 2011). The use of miRNAs as potential biomarkers has been investigated in major depressive disorder (MDD), where miR-16, miR-124, miR-146a/b-5p, miR-24-3p, miR-1202 and miR-135a have been proposed as potential biomarkers for diagnosis and antidepressant treatment efficacy. These findings suggest that the “fingerprint” of circulating miRNAs could potentially be used in the clinic for diagnosis and treatment assignment (Dwivedi, 2014). Additionally, studies suggest that the mode of action of antidepressants may be mediated, at least partially, by miRNAs (Dwivedi, 2014). Patient-derived stable cell lines from blood samples can potentially serve as tools for screening responses to drugs as part of a personalized medicine approach. Replication studies using larger cohorts are needed to verify the validity of these findings and to identify additional relevant miRNAs (Dwivedi, 2014).

Their potential as clinical targets in various medical conditions has been increasingly investigated, including their relevance as therapeutics for brain disorders. One strategy for inhibiting oncogenes or restoring tumor suppressors involves modulating miRNA expression patterns to abolish or restore miRNA biological function. To achieve loss of miRNA function, there are three approaches: miRNA sponges, antisense oligonucleotides (antagomiRs, antimiRs), and genetic knockouts based on the application of Clustered Regularly Interspaced Short Palindromic Repeats-associated protein 9 (CRISPR/Cas9) genome-editing technologies (Diener et al., 2022). The putative relevance of miRNAs for treating brain disorders is underlined by recent developments in clinical research on glioblastoma, a brain malignancy that is difficult to treat despite available therapies such as surgery, radiotherapy, and chemotherapy. Recent studies have investigated miRNA-loaded nanoparticles in glioblastoma models and reported greater treatment efficiency with combined chemotherapy and miRNA-loaded nanoparticles than with either intervention alone (Sadeghipour et al., 2022).

The established role of miRNAs in brain development, combined with the fact that some psychiatric disorders have a neurodevelopmental origin, further supports the possible involvement of miRNAs in the regulation of different psychopathologies (Wang et al., 2021). Recent evidence suggests that miRNAs may play a role in regulating behavior through several mechanisms. Changes in the expression levels of certain miRNAs following behavioral or pharmacological challenges can lead to subsequent changes in the expression of target genes, directing certain behavioral outcomes. Additionally, miRNAs can act as buffers to keep the levels of their protein targets stable, preventing them from being upregulated to pathological levels in response to a challenge. Understanding the role of miRNAs in psychopathologies could provide insight into the molecular pathways disrupted in these disorders, leading to the development of new therapeutic and diagnostic approaches. As miRNAs are potential clinical targets, their identification as therapeutic agents could offer a much-needed avenue for treating psychiatric disorders (Wang et al., 2021; Dwivedi, 2014; Diener et al., 2022).

Despite advances in oncology, evidence that circulating miRNAs regulate behavior, emotion, or cognition in psychiatric conditions remains limited. A recent animal study implicated septal miR-132 downstream of oxytocin receptor signaling in social fear extinction (Bludau et al., 2024). Circulating miRNAs have also been explored in relation to problematic sexual behavior. Hypersexual disorder (HD) was proposed for DSM-5 (Kafka, 2010; Montgomery-Graham, 2017), whereas compulsive sexual behavior disorder (CSBD) was subsequently included as an impulse-control disorder in ICD-11 (WHO, 2022). These constructs overlap but are not interchangeable; the participants in the present historical cohort were assessed using the proposed HD criteria, and we therefore use HD when referring to the study cohort and CSBD only when discussing the contemporary diagnostic context. In an earlier hypothesis-free study of 60 men with HD and 33 healthy controls, higher methylation was observed at a locus associated with hsa-miR-4456 (miR-4456), and subsequent expression analysis indicated lower peripheral blood-derived miR-4456 in HD (Boström et al., 2020). *In-silico* target prediction implicated neuronal pathways, including oxytocin signaling (Boström et al., 2020), providing biological motivation but not evidence of direct regulation.

Oxytocin has been implicated in adaptive processes relevant to psychological and physiological functioning, including social affiliative behavior (Guastella and Hickie, 2016), stress responses (Takayanagi and Onaka, 2022), long-term memory (Brambilla et al., 2016), and food-intake control (Chen et al., 2021). A meta-analysis of exogenously administered oxytocin, mostly delivered intranasally and often as an adjunct to other pharmacological agents, reported preliminary effects in schizophrenia, affective disorders, and autism; however, the included studies were small, and comparative-effectiveness evidence against recommended treatments was lacking (Peled-Avron et al., 2020). Intranasal oxytocin has also been studied in relation to food intake in non-psychiatric participants (Chen et al., 2021). Proposed mechanisms include interactions with dopaminergic, glutamatergic, and serotonergic signaling (Feifel et al., 2016; McQuaid et al., 2014), but uncertainties remain regarding optimal dosing and the duration and central penetration of intranasally administered oxytocin (Peled-Avron et al., 2020). These limitations support investigation of oxytocin-related biology without implying that peripheral biomarkers are established therapeutic targets.

In a subsequent study from the same research program, baseline peripheral oxytocin was higher in men with HD than in healthy controls and decreased in a treated subgroup (Flanagan et al., 2022). Because the source cohorts overlap, these observations and the present analysis should be regarded as convergent, hypothesis-generating extensions rather than independent replication. The present secondary cross-sectional analysis examined whether baseline peripheral miR-4456 expression and baseline plasma oxytocin were quantitatively associated, while explicitly evaluating diagnostic-group confounding and effect modification.

## 2 Materials and methods

### 2.1 Data sources, initial processing, and study design

This secondary cross-sectional analysis used data and archived baseline samples collected in Stockholm during 2013–2014 under protocols approved by the Regional Ethical Review Board in Stockholm (2010/5:3 and 2013/1335-31/2). All participants provided informed consent, and the study followed the Declaration of Helsinki. The baseline oxytocin source cohort comprised 64 men diagnosed according to the proposed DSM-5 HD criteria and 38 healthy male controls (Flanagan et al., 2022). HD eligibility required age >18 years and stable psychoactive medication for at least 3 months; paraphilic disorders, severe psychiatric comorbidity, and dopamine-agonist use were exclusion criteria. Diagnosis and comorbidity were assessed by clinicians experienced in sexual medicine using the proposed HD criteria and the Mini International Neuropsychiatric Interview 6.0. Controls had no history of psychiatric illness. Clinical characterization included HDSI (Association, 2010), HD:CAS (American Psychiatric Association, 2010), MADRS (Montgomery and Asberg, 1979), SCS (Kalichman and Rompa, 1995), and CTQ (Bernstein and Fink, 1998). The analysis was not preregistered. Its hypothesis was biologically informed by earlier findings in an overlapping research program and should not be interpreted as independent replication.

### 2.2 Blood sample collection and assessments

The source studies used different documented collection conditions. The miRNA source study reported morning, non-fasting EDTA-blood collection (Boström et al., 2020), whereas the oxytocin source study reported morning fasting blood collection at approximately 08:00 (Flanagan et al., 2022). Samples were processed according to the respective source-study protocols and stored at −80 °C until analysis. The retained documentation does not establish that the two analytes were obtained from the same blood draw or aliquot. Only pretreatment baseline oxytocin measurements were used in the present association analyses. Post-treatment samples from the CBT subgroup were not used because post-treatment miR-4456 was not measured and the present research question was cross-sectional.

### 2.3 Measurement of hsa-miR-4456

#### 2.3.1 RNA extraction

After thawing, 800 μL of EDTA blood-derived material was centrifuged for 10 min at 1,900 × g and then for 10 min at 16,000 × g at 4 °C. RNA was extracted from 200 μL of the resulting supernatant using the miRNeasy Serum/Plasma Kit (Qiagen, Hilden, Germany) according to the manufacturer's instructions (Boström et al., 2020). The source study reported erythrocyte hemolysis that prevented complete separation of plasma from the corpuscular blood fraction. Accordingly, the miR-4456 measurement is described here as peripheral EDTA blood-derived rather than as a validated cell-free plasma miRNA measurement; the source report stated that all samples were affected (Boström et al., 2020).

#### 2.3.2 Reverse transcription and quantitative real-time PCR

Ten nanograms of extracted RNA was reverse-transcribed using the TaqMan MicroRNA Reverse Transcription Kit and stem-loop assays for miR-708-5p and miR-4456. Reverse transcription was performed at 16 °C for 30 min, 42 °C for 30 min, and 85 °C for 5 min. Quantitative PCR used 0.67 μL cDNA and 9.3 μL Universal Fast Master Mix including miRNA-specific primers, with 95 °C for 20 s followed by 40 cycles of 95 °C for 1 s and 60 °C for 20 s on a ViiA 7 Real-Time PCR System. Measurements were run in triplicate and normalized to U6 small nuclear RNA (Boström et al., 2020). miR-708-5p was measured in the source study but was not part of the biologically specified miR-4456–oxytocin question examined here. The archived documentation available for this revision did not contain catalog/assay identifiers, Ct distributions or prespecified Ct thresholds, the exact Ct-to-expression calculation, exogenous spike-in recovery data, or an experimental evaluation of U6 stability in this cohort; these limitations are reported explicitly below.

##### 2.3.2.1 Sample exclusion considerations

The earlier epigenomic source cohort comprised 93 participants (60 HD and 33 controls). After source-study processing and quality-control exclusions, the expression dataset comprised 87 participants (Boström et al., 2020). Four miR-4456 expression observations were subsequently identified by visual boxplot inspection and excluded in that source study, leaving 83 participants with miR-4456 data (55 HD and 28 controls). No numerical outlier threshold, excluded values or group membership, or documentation establishing blinding to oxytocin was retained. These exclusions preceded the present association analysis. The archived RData file available for the revision contains only the filtered observations; consequently, the four excluded measurements could not be restored for the requested inclusion sensitivity analysis. Of the 83 miR-4456 observations, 74 had a paired baseline oxytocin value for the submitted pooled analysis. One of these did not have usable group coding in the archived merged analysis object, leaving 73 complete cases (51 HD and 22 controls) for group-adjusted and interaction models. Because the source datasets overlap but are not a single conventional attrition chain, counts are shown by source dataset and analysis set in Table 1.

**Table 1:** Participant availability and analysis sets.

Dataset or analysis set	HD	Control	Total	Interpretation
Baseline oxytocin source cohort	64	38	102	Baseline, pretreatment oxytocin cohort
Earlier epigenomic source cohort	60	33	93	Partially overlapping source cohort; not a simple subset of all 102
miRNA expression dataset after source processing exclusions	—	—	87	Source expression dataset before exclusion of four miR-4456 observations
miR-4456 after source-study outlier exclusion	55	28	83	Four miR-4456 observations had already been excluded in the source study
Paired baseline miR-4456 and oxytocin	—	—	74	Submitted pooled robust model
Complete cases with group status	51	22	73	Revised group-adjusted and interaction models

Source datasets overlap but are not a single conventional attrition chain. Group counts for the 87-person intermediate expression dataset and the 74 paired observations could not be reconstructed from the retained files. One paired record did not enter the archived models requiring group status, and the retained documentation does not establish the reason.

### 2.4 Measurement of oxytocin levels in blood plasma

Baseline plasma oxytocin was measured by radioimmunoassay at the Clinical Neurochemistry Laboratory, Sahlgrenska University Hospital, Mölndal, Sweden, using antibody RRID:AB_2894933, as described previously (Flanagan et al., 2022). The lower limit of detection was 5 pM. Intra- and interassay coefficients of variation were 14% and 8% at 12 pM, 10% and 15% at 31 pM, and 12% and 10% at 60 pM, respectively. The source documentation available for this revision did not provide participant-level extraction recovery, batch allocation, duplicate-discordance rules, technician blinding, or a complete freeze–thaw history. These unrecorded assay features and the moderate coefficients of variation limit claims about measurement precision.

### 2.5 Statistical analysis

Analyses were performed in R 4.0.3. Shapiro–Wilk tests indicated departures from normality for baseline oxytocin and miR-4456. Consistent with the submitted analysis code, miR-4456 expression alone was rank-based inverse-normal transformed using Blom's method; oxytocin remained on its original pmol/L scale. Accordingly, each regression coefficient represents the difference in plasma oxytocin (pmol/L) per one-unit increase in Blom-transformed miR-4456 expression.

The originally submitted model was a pooled robust MM-regression of baseline oxytocin on miR-4456 using robustbase with the KS2014 setting. That unadjusted model used 74 paired measurements. To address diagnostic-group confounding, the revised main model included diagnostic group as a covariate (oxytocin ~ miR-4456 + group) in 73 complete cases. A secondary robust model included a miR-4456-by-group interaction to formally test effect modification. Group-specific slopes and their confidence intervals were calculated as linear combinations of coefficients from the interaction model using its fitted covariance matrix. The model was deliberately parsimonious because only 73 complete paired observations were available and the archived merged analysis file did not include participant-level age, BMI, smoking, medication, endocrine, depressive-symptom, or trauma variables. A larger adjustment model would therefore have been unreproducible from the available file and vulnerable to overfitting. Confidence intervals are two-sided 95% intervals, and all reported *p* values are two-sided.

*Post hoc* sensitivity analyses comprised a group-adjusted ordinary least-squares model with HC3 heteroskedasticity-consistent standard errors; 10,000 group-stratified non-parametric bootstrap resamples of the group-adjusted robust coefficient; a partial Spearman correlation controlling for diagnostic group, evaluated with 10,000 outcome permutations within group; and leave-one-out refitting of the group-adjusted robust model after omitting each participant in turn. Bootstrap percentile intervals and the proportion of bootstrap coefficients below zero were treated as stability summaries rather than confirmatory probabilities.

Model assumptions and influence were assessed using residual-vs.-fitted, quantile–quantile, residual-vs.-miR-4456, and robust-weight plots. The residual-vs.-miR-4456 plot did not show pronounced systematic curvature; given the modest sample size, no higher-order exposure terms were added. During revision, OpenAI Codex (GPT-5; OpenAI, accessed September 2026) was used to assist with code review and language and document preparation. All code, statistical outputs, citations, and statements were independently reviewed by the authors, who take full responsibility for the work.

## 3 Results

### 3.1 Baseline characteristics of data

The baseline oxytocin source cohort included 64 men with HD and 38 healthy controls; the earlier epigenomic source cohort included 60 men with HD and 33 controls; and the source-study expression dataset included 87 participants after processing exclusions and 83 participants (55 HD and 28 controls) after exclusion of four miR-4456 observations (Table 1). The submitted pooled association analysis contained 74 paired baseline biomarker measurements, and 73 participants (51 HD and 22 controls) contributed to analyses requiring diagnostic-group status. In the previously published source cohorts, the HD group had higher MADRS and CTQ scores, whereas age and the reported endocrine measures did not differ significantly (Boström et al., 2020; Flanagan et al., 2022). Participant-level clinical covariates for the exact 73-person merged analysis set were not present in the archived analysis object, precluding a new complete-case baseline-covariate table or multivariable adjustment beyond group.

### 3.2 Association between miR-4456 expression and baseline plasma oxytocin

In the submitted pooled robust model (*n* = 74), a one-unit higher Blom-transformed miR-4456 value was associated with 4.039 pmol/L lower plasma oxytocin (β = −4.039, 95% CI −6.149 to −1.930; *p* < 0.001; Table 2 and Figure 1). In the complete-case pooled model restricted to participants with available diagnostic-group information (*n* = 73; Figure 2A), the corresponding unadjusted estimate was −4.046 (95% CI −6.159 to −1.934; *p* < 0.001). After adjustment for diagnostic group, the estimate was attenuated to −1.522 (95% CI −3.376 to 0.333; *p* = 0.106). Thus, the revised main model retained a negative point estimate, but its confidence interval included no association after accounting for the marked between-group difference in plasma oxytocin.

**Table 2:** Robust regression estimates for the association between miR-4456 expression and plasma oxytocin.

Analysis	Estimand	*n*	β	95% CI	*p* value
Submitted pooled robust model	Overall slope	74	−4.039	−6.149 to −1.930	< 0.001
Complete-case pooled robust model	Overall slope	73	−4.046	−6.159 to −1.934	< 0.001
Group-adjusted robust model	Common within-group slope	73	−1.522	−3.376 to 0.333	0.106
Interaction model	Control-specific slope	22	−0.613	−3.693 to 2.467	0.696
Interaction model	HD-specific slope	51	−2.223	−4.514 to 0.069	0.057
Interaction model	HD minus control slope difference	73	−1.609	−5.517 to 2.298	0.414

Oxytocin is expressed in pmol/L; miR-4456 is Blom-transformed. β is the difference in oxytocin per one-unit increase in transformed miR-4456. The group-adjusted model is the revised main analysis. Group-specific estimates and the interaction were calculated from the interaction model and are interpreted cautiously.

**Figure 1:**
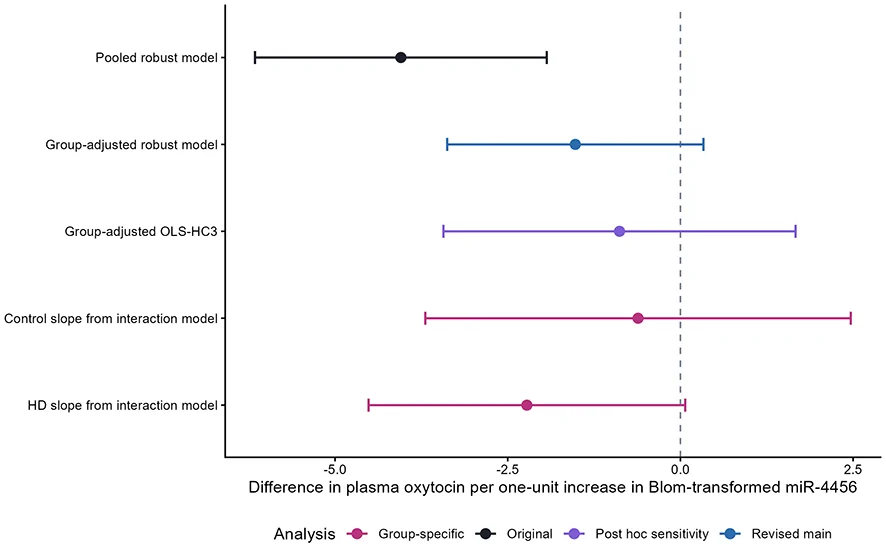
Regression estimates across the submitted, revised, and group-specific models. Points are regression coefficients and horizontal bars are 95% confidence intervals. The dashed vertical line indicates no association. The complete-case pooled robust model (*n* = 73) is shown for comparison; the group-adjusted robust model is the revised main analysis. The OLS-HC3 and group-specific estimates are *post hoc* or reviewer-requested sensitivity estimates.

**Figure 2:**
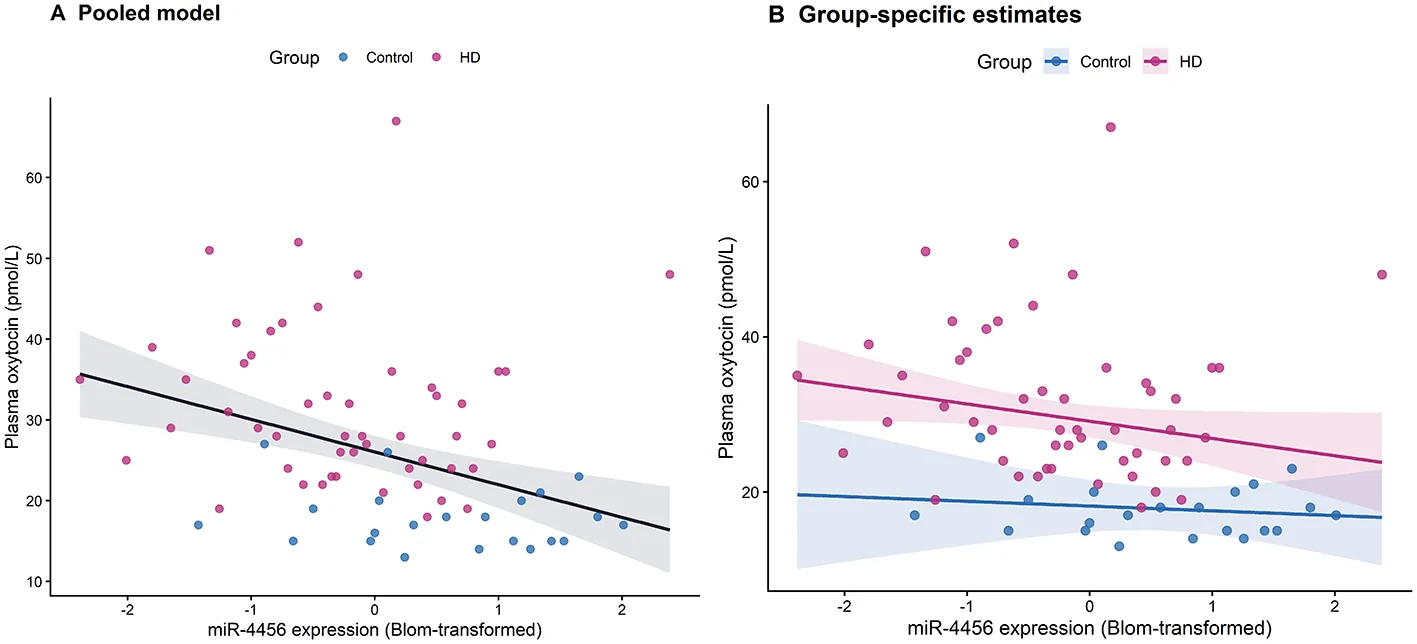
Pooled and group-specific associations between miR-4456 expression and plasma oxytocin. **(A)** shows the complete-case pooled association (*n* = 73), with points colored by diagnostic group. **(B)** shows group-specific fitted slopes from the interaction model. miR-4456 alone was Blom-transformed; oxytocin remains in pmol/L. Shaded areas are 95% confidence bands. HD, hypersexual disorder.

A robust interaction model was used to examine whether the association differed between diagnostic groups (Figure 2B and Table 2). The estimated slope was −0.613 pmol/L per unit increase in Blom-transformed miR-4456 among controls (95% CI −3.693 to 2.467; *p* = 0.696) and −2.223 among participants with HD (95% CI −4.514 to 0.069; *p* = 0.057). The miR-4456-by-group interaction was not statistically significant (β = −1.609, 95% CI −5.517 to 2.298; *p* = 0.414). Accordingly, the data did not provide evidence that the association differed by diagnostic group, although the group-specific estimates were imprecise and the study was not adequately powered to establish or exclude effect modification.

*Post hoc* sensitivity analyses showed consistently negative point estimates but limited precision (Supplementary Table S1, and Supplementary Figures S1–S4). OLS with HC3 standard errors gave β = −0.881 (95% CI −3.431 to 1.668; *p* = 0.493). A group-stratified robust bootstrap yielded a 95% percentile interval of −3.373 to 0.613, with 92.0% of resampled slopes below zero. The group-adjusted partial Spearman correlation was ρ = −0.201 (two-sided stratified permutation *p* = 0.089). Across 73 leave-one-out robust regressions, the estimates ranged from −1.856 to −1.260 and were uniformly negative. Because these highly overlapping leave-one-out estimates are not statistically independent, no binomial *p* value was calculated. This analysis indicates that the negative point estimate was not reversed by omission of any single retained participant. Graphical diagnostics did not show pronounced systematic curvature. These *post hoc* analyses indicate directional stability of the negative point estimate but do not constitute independent confirmation of the association.

## 4 Discussion

This secondary cross-sectional analysis provides the first direct examination of peripheral miR-4456 and plasma oxytocin measurements in the same human participants. The submitted pooled analysis showed an inverse association. Adjustment for diagnostic group substantially attenuated the coefficient and widened its confidence interval across zero, indicating that between-group separation accounts for part of the pooled relationship. The revised results therefore do not establish a group-independent association, but they define a specific paired-biomarker observation that merits independent study.

The scientific relevance of this observation lies in how it connects several previously separate findings. Earlier work identified hypermethylation at a miR-4456-associated locus, lower peripheral blood-derived miR-4456 expression in hypersexual disorder, and enrichment of predicted miR-4456 targets in oxytocin-related pathways (Boström et al., 2020). A subsequent study in the same research program found higher baseline plasma oxytocin in men with hypersexual disorder (Flanagan et al., 2022). Each observation alone is indirect. Demonstrating that miR-4456 and oxytocin covary when measured within the same individuals provides a new empirical link between the molecular and neuroendocrine findings. Because the cohorts overlap, this is a convergent extension rather than an independent replication, but the paired measurements address a question that the earlier studies could not answer.

The group-specific results refine the interpretation. The estimated slope was more negative in the HD group than among controls, but both confidence intervals were wide and the formal interaction test was not significant. The control subgroup contained only 22 participants and was particularly imprecise. Accordingly, the study was not adequately powered to establish or exclude effect modification, and the observed numerical difference between slopes should not be interpreted as evidence of a stronger association in HD. Adequately powered replication is needed to determine whether any association is present within either group.

The *post hoc* sensitivity analyses add information about stability without converting the result into independent confirmation. Ninety-two percent of stratified bootstrap estimates were negative, the group-adjusted partial Spearman coefficient was −0.201, and every leave-one-out robust-regression coefficient remained negative. The uniformly negative leave-one-out coefficients support influence stability: no single retained participant determined or reversed the direction of the group-adjusted estimate. Because the leave-one-out samples overlap extensively, this finding should not be interpreted as 73 independent replications or as additional confirmatory significance. At the same time, the bootstrap interval included zero and the two-sided permutation *p* value was 0.089. Taken together, these analyses show that the negative point estimate was not driven by a single retained observation or one regression specification, while its magnitude and statistical certainty remain limited after accounting for group.

The study has several strengths. Both biomarkers were available from the same clinically characterized participants, both source protocols used morning sampling, and the hypothesis followed a biologically coherent sequence of epigenetic, expression, pathway-prediction, and hormonal observations. The revision also separates the pooled and group-adjusted estimands, reports group-specific uncertainty, and includes non-supportive as well as supportive sensitivity analyses. These features make the dataset useful for hypothesis generation despite its modest size.

The study has important limitations. The analysis was not preregistered, reused partially overlapping source cohorts, and included only men. Clinical covariates for the exact paired set were unavailable, leaving potential confounding by medication, symptoms, trauma, lifestyle, or metabolic factors. Four miR-4456 observations had been excluded in the source study using visual boxplot inspection and could not be restored from the filtered archive. The miRNA and oxytocin source studies documented different fasting conditions, and the retained records do not establish that both analytes came from the same draw or aliquot. The source miRNA material was affected by erythrocyte hemolysis and included plasma and corpuscular-blood components; cohort-specific U6 stability, spike-in recovery, platelet contamination, and complete batch and freeze–thaw information were unavailable. Oxytocin assay variability and uncertain correspondence between peripheral and central concentrations further limit precision and mechanistic interpretation (Tabak et al., 2023). These limitations preclude causal or central nervous system claims and require independent replication with improved preanalytical control.

The results define a focused next step. Independent studies should obtain paired miR-4456 and oxytocin measurements under harmonized collection conditions, with validated plasma-miRNA normalization, hemolysis and platelet quality control, complete clinical covariates, and sufficient power for within-group and interaction estimates. Mechanistic studies can then test whether experimental manipulation of miR-4456 changes validated oxytocin-pathway targets, oxytocin synthesis or release, or related signaling in relevant cells and animal models. Such work is necessary before miR-4456 can be described as a regulator or therapeutic target, but the present paired observation provides a specific rationale for undertaking it.

## 5 Conclusion

This study is the first human investigation to directly examine paired peripheral miR-4456 and plasma oxytocin measurements in a clinical-control cohort. The inverse pooled association was attenuated after adjustment for diagnostic group, and the available sample could not determine whether an association is present within either group or differs between groups. The findings therefore do not establish that miR-4456 regulates oxytocin, but they connect prior epigenetic, expression, pathway-prediction, and neuroendocrine observations into a specific and experimentally testable hypothesis. Independent replication and direct mechanistic studies are warranted to determine whether miR-4456 has a functional role in oxytocin-related biology.

## Data Availability

The datasets presented in this article are not readily available because de-identified data and the analysis code underlying the reported results are available from the corresponding author on reasonable request, subject to ethical approval and applicable data-protection requirements. Requests to access the datasets should be directed to Jussi Jokinen, jussi.jokinen@umu.se.
