# Supplementary material for: Association between peripheral hsa-miR-4456 expression and plasma oxytocin in men with hypersexual disorder and healthy controls: a secondary cross-sectional analysis
Source: Front Neurosci. 2026 Oct 14;20:1946302. doi: 10.3389/fnins.2026.1946302 (PMC13713500; doi:10.3389/fnins.2026.1946302)
Supplement: Supplementary file 1 [file Supplementary_file_1.docx]

**Supplementary Material**

**Supplementary Table S1. Post hoc sensitivity analyses**

| **Analysis** | **Result** | **p value** | **Classification** |
| --- | --- | --- | --- |
| Robust group-adjusted regression | β = −1.52 (95% CI −3.38 to 0.33) | 0.106 | Main revised analysis |
| OLS with HC3 standard errors | β = −0.88 (95% CI −3.43 to 1.67) | 0.493 | Post hoc specification sensitivity |
| Stratified robust bootstrap | 95% CI −3.37 to 0.61; 92.0% negative | — | Post hoc precision sensitivity |
| Partial Spearman permutation | Partial ρ = −0.201 | 0.089 | Post hoc two-sided sensitivity |
| Robust leave-one-out stability | β range −1.86 to −1.26; 100% negative | — | Post hoc influence sensitivity |
| Outlier-inclusion sensitivity | Not reproducible from the archived filtered file | — | Reviewer-requested; limitation reported |

Note: All sensitivity analyses added after peer review are post hoc. The bootstrap proportion below zero is a stability summary, not a confirmatory probability. All reported p values are two-sided.

**Supplementary Figure S1. Stratified robust bootstrap distribution**

The blue line marks the observed group-adjusted robust estimate and the dashed line marks zero. The percentile 95% interval crosses zero.

**Supplementary Figure S2. Stratified permutation null distribution for the partial Spearman statistic**

The magenta vertical line shows the observed group-adjusted partial Spearman correlation. Outcome values were permuted within diagnostic group; the displayed p value is two-sided.

**Supplementary Figure S3. Leave-one-out stability of the group-adjusted robust coefficient**

The solid line is the full-sample group-adjusted estimate and the dashed line indicates no association. Point color denotes the omitted participant's group.

**Supplementary Figure S4. Robust-model diagnostic plots**

These plots are presented for diagnostic transparency. They include residual-versus-fitted, quantile–quantile, residual-versus-miR-4456, and robust-weight plots. No pronounced systematic curvature was evident in the residual-versus-miR-4456 plot.
